# Acoustic Emissions in 3D Printed Parts under Mode I Delamination Test

**DOI:** 10.3390/ma11091760

**Published:** 2018-09-18

**Authors:** Claudia Barile, Caterina Casavola, Alberto Cazzato

**Affiliations:** Dipartimento di Meccanica, Matematica e Management (DMMM), Politecnico di Bari, Viale Japigia 182, 70126 Bari, Italy; claudia.barile@poliba.it (C.B.); alberto.cazzato@poliba.it (A.C.)

**Keywords:** acoustic emission, double cantilever beam, fused deposition modelling, Mode I fracture mechanics, orthotropic materials

## Abstract

This paper applies an innovative approach based on the acoustic emission technique to monitor the delamination process of 3D parts. Fused deposition modelling (FDM) is currently one of the most widespread techniques for additive manufacturing of a solid object from a computer model. Fundamentally, this process is based on a layer-by-layer deposition of a fused filament. The FDM technique has evolved to the point where it can now be proposed, not only as a prototyping technique, but also as one applicable to direct manufacturing. Nonetheless, a deeper comprehension of mechanical behavior and its dependence on process parameters must include the determination of material properties as a function of the service load. In this work, the effects of extrusion temperature on inter-layer cohesion are studied using a method employing a double cantilever beam (DCB). The ASTM D5528 standard was used to determine the delamination energy, *G_I_*. In addition, the acoustic emission technique was employed to follow the delamination process during testing. Finally, a Charge-Coupled Device (CCD) camera and a calibrated grid was employed to evaluate crack propagation during testing.

## 1. Introduction

Fused deposition modelling (FDM) is currently one of the most widespread additive manufacturing techniques at both commercial and business levels. It can be used to create a final prototype from a Computer-Aided Drafting (CAD) model and represents an interesting tool in many fields, spanning those of biomedical, aerospace, heritage, and building [1,2]. Several low-cost 3D printers exist, such as Maker.Bot, Ultimaker, RepRap, Cube, etc., that make this technology accessible at both home and the office. In principle, the idea behind the FDM process is that of a layer-by-layer deposition of a feedstock wire which is heated to melting point. Partially melted filament is then extruded and deposited through a numerically controlled nozzle [1]. Once deposited, the material solidifies over the previous layer and deposition of the following layer is then started after raising the nozzle. Due to both this layer-by-layer building process and the intrinsic orientation of the deposited material, parts manufactured by FDM have an orthotropic behavior similar to that of a laminate orthotropic structure [3,4,5]. Initially, only polylactic acid (PLA) and acrylonitrile-butadiene-styrene (ABS) could be processed by FDM. However, many other materials can now be utilized in this process, ranging from ceramic to metal, and short fiber composites to bio-resorbable polymers (PCL). Compared to ABS, PLA has higher mechanical resistance and a lower coefficient of thermal expansion. The latter characteristic increases the printability of the materials because warping effects, residual stresses and de-layering phenomena are reduced [6,7,8,9,10].

One of the keys to the success of FDM technology is that it allows the creation of small quantities and unique parts having customized geometries and materials. Technological evolution of this technique in recent years has advanced this approach from a prototyping method to a rapid manufacturing method [11]. However, progress in terms of knowledge of mechanical behavior and its dependence on the proper choice of parameter settings is still required. Many recent studies have been devoted to analyzing the variation of mechanical properties as a function of printing settings and on the effect of raster orientation [3,12,13,14,15]. However, only a few papers [16,17] have investigated how process parameters affect inter-layer bonding strength. [16] prove that 90° rectangular testing specimens (ASTM D3039 Tensile test) could be used to investigate this property. In addition, in the same paper, the advantage of depositing shifted layers was noted as having effects on surface roughness. In [17], the effect of the layer design was investigated for the case of a PLA-printed object in terms of inter-layer and intra-layer cohesion. Analysis is, however, limited to the single PLA material. In this paper, the influence of the extrusion temperature on inter-layer cohesion is investigated for the case of ABS-printed parts. Mechanical tests were performed employing a double cantilever beam (DCB) specimen according to ASTM D5528 [18]; acoustic emissions (AE) were recorded during the tests. Specimens obtained using three extrusion temperatures (i.e., 220 °C, 230 °C, and 240 °C) were tested and the delamination energy *G_I_* was evaluated.

AE has not previously been applied to this class of materials; it was introduced with the aim of obtaining extra information about the behavior of the part during the test. It allows monitoring of the test, in which identification of variations in recorded acoustic events can be traced to nucleation and propagation of cracks inside the material. The approach is a passive one: no sound wave is produced externally, but detection is limited to the acoustic events produced directly inside the sample as a consequence of the presence of an active defect. AE has been proven to be capable of detecting sudden motions in stressed parts at a large scale, such as in earthquake events, and at a small scale, such as in the case of dislocation motion. The analysis of the evolution of these types of events has allowed researchers to develop appropriate mathematical relationships that have been successfully tested in several industrial applications [19,20,21]. Appropriate indicators of acoustic activity have been identified, such as number of hits, amplitude, waveform features, energy, etc. Taking advantage of statistical methods, these indicators can be analyzed and combined to provide information about the material’s behavior [22,23,24,25,26]. This method was successfully applied to metallic materials [27,28,29], and glass fiber reinforced polymer (GFRP) [30,31]. Only a few results are available in the literature for carbon fiber reinforced polymer (CFRP) and FDM parts.

## 2. Materials and Methods

### 2.1. Mode I DCB Test

**Figure 1:**
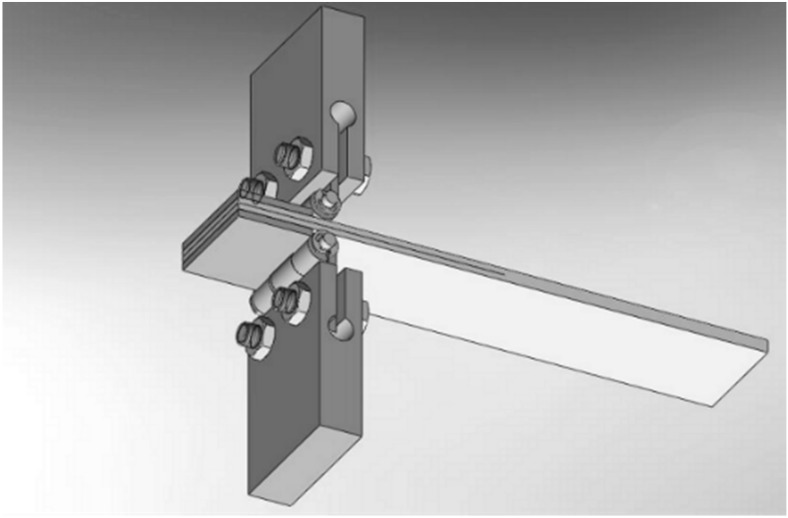
Mode I delamination test: schematic of the equipment.

**Figure 2:**
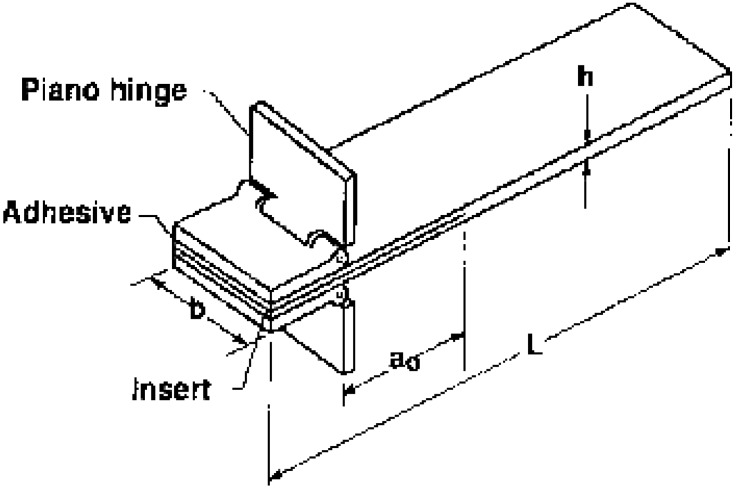
Specimen geometry according to ASTM D5528.

Three different extrusion temperatures were tested, namely, 220 °C, 230 °C and 240 °C. For each temperature, 3 samples were prepared and tested for a total of 9 DCB specimens. Samples had a 25 mm × 3 mm section and a length of 125 mm. A Kapton tape was inserted at the midplane of the specimen during manufacturing so that it acted as an initiation site for delamination. The thickness of the tape was less than 13 μm and its length was 63 mm. An overall schematic is displayed in Figure 1. Figure 2 and Table 1 report the geometry and the dimensions of the specimens according to the ASTM D5528 standard [18], respectively.

Following the ASTM D5528-01 standard, a unidirectional printing approach was adopted, in which the deposition of the infill occurred parallel to the longitudinal axis of the sample. An Ultimaker 2+ equipped with a 0.4 mm nozzle was employed to manufacture the specimens. The main process parameter settings for this experiment are displayed in Table 2. The bed temperature was 90 °C and extrusion temperatures were set as indicated above. Specimens were manufactured with the thickness perpendicular to the base platform. The air gap indicated in Table 2 is the distance between near beads in the same layer, while the bead width and the layer thickness represent the width and height of the filament, respectively. The number of contours represents the number of deposited edges before initiating the filling of the internal parts.

**Figure 3:**
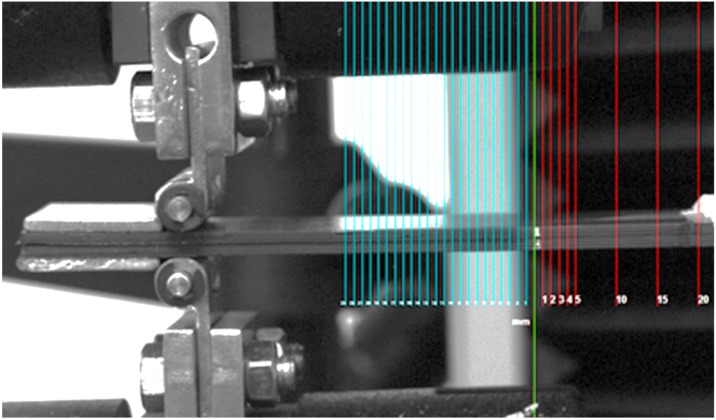
Acquired image of the specimen showing the overlaid digital grid.

Mechanical tests were performed with a displacement rate equal to 1 mm/min using the electro-mechanical Instron 3343 testing machine. In contrast to ASTM D5528, a digital calibrated grid (rather than a manual grid) was adopted during the test. The grid was superimposed onto the images recorded during the test by an Allied Marlin CCD camera (Figure 3). A frame rate of acquisition equal to 12.75 frames/s was set to follow the entire test. To facilitate visualization, the grid was colored in two ways: blue lines corresponded to the position of the insert while the red lines were used to evaluate damage propagation. According to [18], the extension of the blue grid, indicated by *a*_0_, indicated the starting delamination length.

Delamination in a part displaying a linear elastic material is associated with a strain energy release rate (*G*) that can be given by the following equation:

(1) $$G=-\frac{dU}{B\;da}$$

where *a* is the crack length, *B* is the specimen width and *U* is total potential energy of the specimen.

For the case of the Mode I DCB test, the strain energy release rate can be calculated by using the formula derived from beam theory (*BT*):

(2) $$G_{I}^{BT}=\frac{3\;P\;\delta }{2\;B\;a}$$

where *P* is the maximum load and *δ* is the deflection in correspondence of the load. This theory may be corrected by considering the changing compliance at the end of the crack that causes both rotation and deformation of the specimen, modifying the distance between the applied load and the end of the crack. This correction, known as modified beam theory (*MBT*), reduces $G_{I}^{BT}$ toughness as follows:

(3) $$G_{I}^{MBT}=\frac{3\;P\;\delta }{2B\;\left(a+\Delta \right)}$$

where Δ may be determined experimentally by generating a least squares plot of the cube root of compliance (C^1/3^), as a function of delamination length.

**Figure 4:**
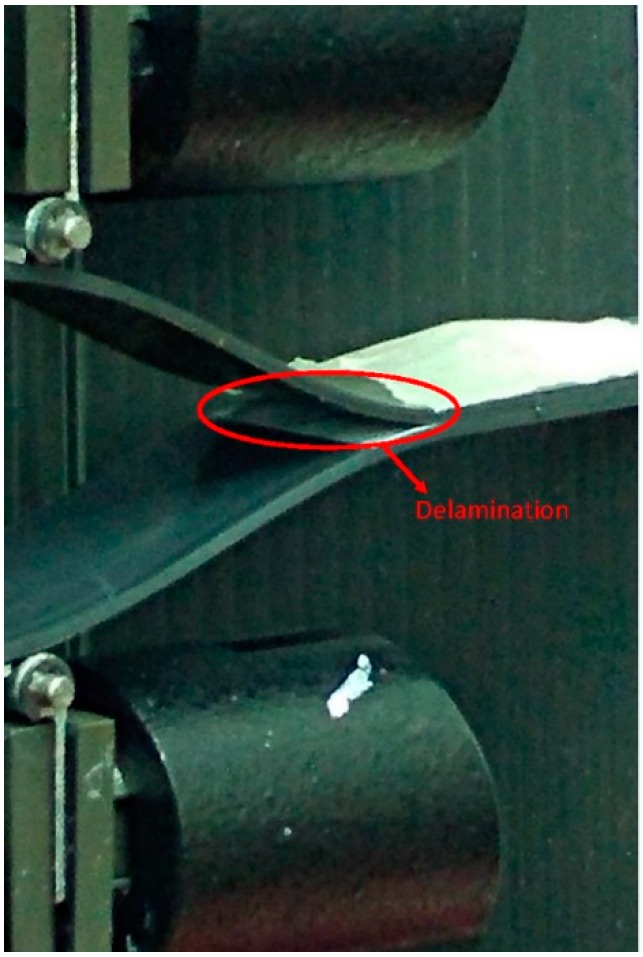
Crack opening during delamination test.

In Figure 4, an image of the delaminated specimen is shown. Crack opening and propagation at the end of the insert is clearly visible.

### 2.2. Acoustic Emission

A piezoelectric sensor was attached to the surface of the sample in order to detect acoustic signals generated during the test. A silicone grease was used in order to guarantee an optimal coupling between the sensor and the sample. In particular, a Pico sensor (5 mm × 4 mm) was used and placed 10 mm from the border of each sample. A Pico sensor is characterized by an operating frequency range between 200 kHz and 750 kHz, with a resonance frequency equal to 250 kHz. It can be used in a temperature range from −65 °C to 177 °C and its peak sensitivity is 54 dB. The sensor was connected to a 20/40/60-AST preamplifier. For these experiments, the gain was set to 40 dB. The amplified signal was sent to a PCI-2 acquisition board, while the signals were analyzed by AEWin software. This allows extraction of the key features of acoustic signals, such as amplitude, energy, hits and counts. Acquisition frequency was maintained at 100 kHz for all the tests, while the acquisition amplitude threshold was maintained at 35 dB in order to achieve efficient noise reduction and good signal detection simultaneously.

## 3. Results and Discussion

**Figure 5:**
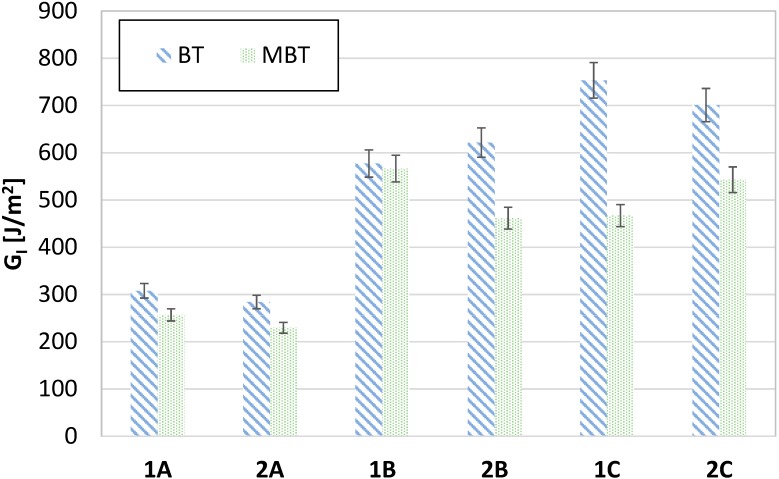
*G_I_* values calculated by Beam Theory (BT) and Modified Beam Theory (MBT).

From examination of the results, differences can be observed for the three analyzed temperatures of the extrusion nozzle for *G_I_* toughness calculated according to beam theory (BT) and the modified beam theory (MBT); MBT corrects BT by considering the changing compliance at the end of the crack, which causes both rotation and deformation of the specimen. The crack opening and detected acoustic signals also displayed differences. In Figure 5, the letters A, B, and C refer to the three extrusion temperatures: 220 °C, 230 °C and 240 °C, respectively. The specimens are named 1, 2, and 3. For each group, three samples were tested. However, only two specimens are reported in Figure 5. The first was neglected because it was used for calibrating the digital grid; therefore, no information could be inferred for crack length and only AE data could be collected. From Figure 5, it is possible to infer a reduction of *G_I_* calculated by MBT relative to BT. Figure 5 also shows the errors bar of the results. This confirms that large deformations occurred for FDM specimens, as expected, especially if created at high temperature; in this case, correction of *G_I_* is mandatory for comparison with CFRP specimens [32,33]. Indeed, the most significant reduction in *G_I_* toughness was observed for the group manufactured at 240 °C.

**Figure 6:**
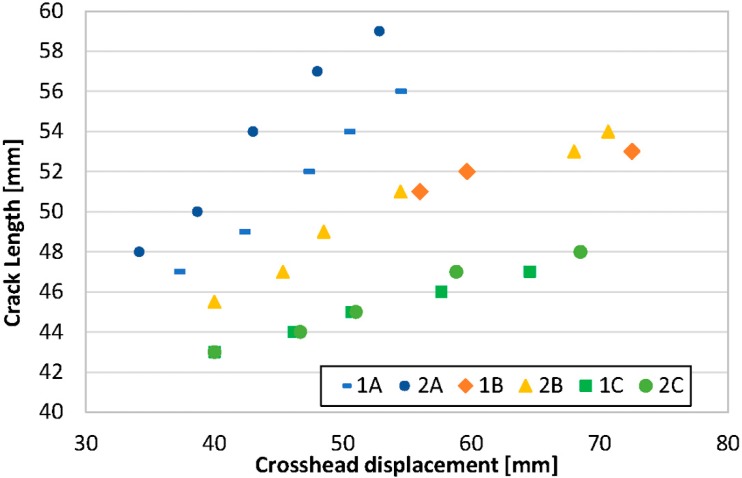
Crack length vs. crosshead displacement.

Figure 6 shows the plot of crack length vs. crosshead displacement. It is clear that the extrusion temperature has an effect on crack behavior. For the group A specimens (220 °C), the highest values of crack length correspond to the lowest values of crosshead displacement; for the specimens belonging to group B (230 °C), intermediate values of crack lengths are observed to be scattered over a wide interval of crosshead displacements; and, for the specimens belonging to group C (240 °C), the lowest values of crack length were observed to be in correspondence with a wide range of crosshead displacements. This behavior can be theorized to be the consequence of different cohesion between adjacent strands depending on the extrusion temperatures [17]. It is reasonable to say that if temperature is increased, the fusion of the layers is increased and the highest values of *G_I_* toughness are obtained.

**Figure 7:**
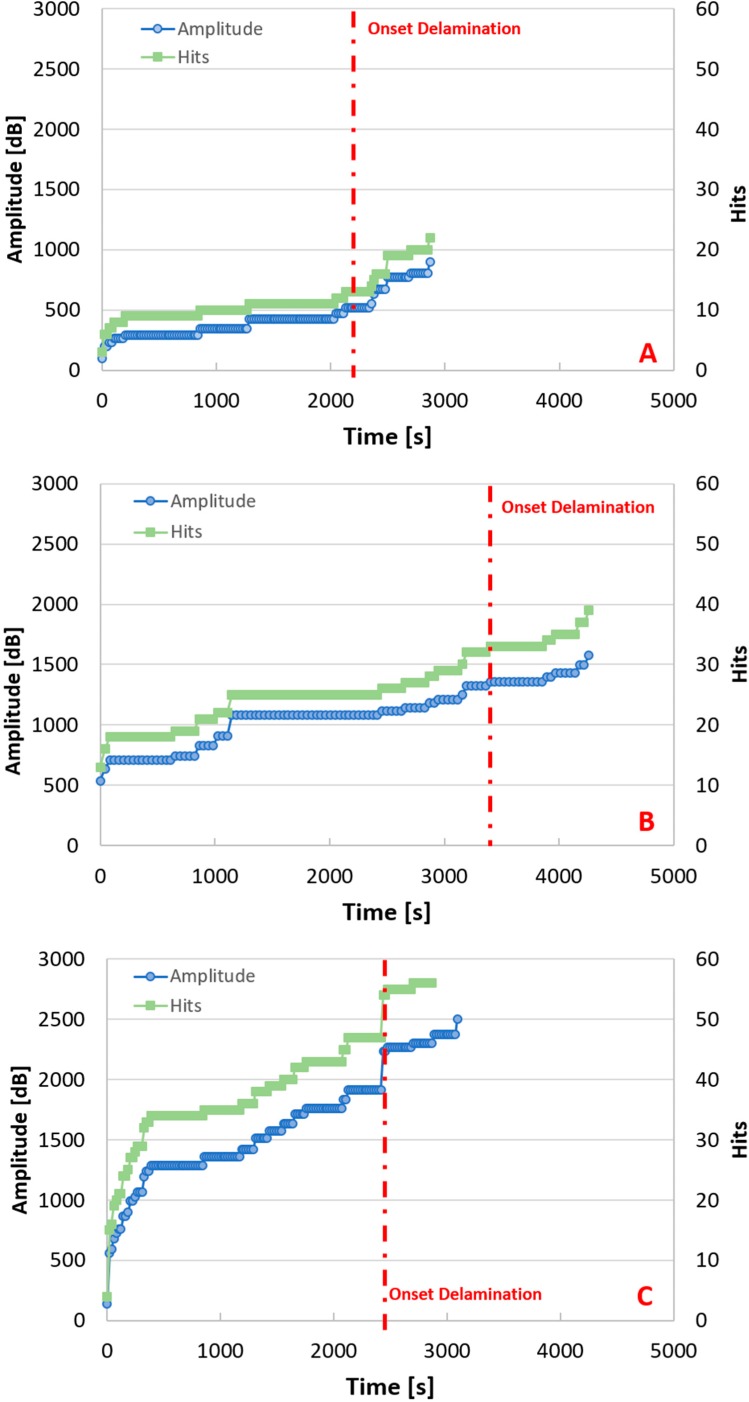
Acoustic emissions recorded: (**A**) refers to Group A specimen (220 °C); (**B**) refers to Group B specimen (230 °C); (**C**) refers to Group C specimen (240 °C).

Furthermore, analysis of the AE data shows some differences distinguishing the three temperatures. Acoustic data are presented in Figure 7. Charts in Figure 7 refer to amplitude, which represents the largest voltage peak in the acoustic emission signal waveform (expressed in decibels), and hits, which is the number of acoustic signals detected and measured on a channel during the tests. It is important to underline that the error bars associated with the amplitude are not included in the graphs, since the most useful and significant parameter in damage propagation monitoring is the evolution of the trend of the amplitude of the signals recorded, rather than its variability. From Figure 7 it can be inferred that the number of hits and amplitude change as a function of the extrusion temperature. Specifically, the number of hits increases if the extrusion temperature is increased. Moreover, the highest amplitudes are observed for the samples of group C (manufactured at the highest temperature, 240 °C). From a general point of view, it is possible to conclude that the highest acoustic activity relates to highest temperature. In conjunction with the previous results, this behavior suggests an increment of the *G_I_* toughness. If temperature increases, in fact, specimens become more compact and, as a consequence, they produce more acoustic signals for the same applied load.

Interesting observations can be drawn in connection with the predictive capacity of AE if compared to visual inspection performed during the Mode I tests. The dashed line in Figure 7 indicates the onset of delamination. The initial time of delamination was different in the three groups but the predictive capability does not change. The tests were undertaken by controlling displacement; in that way the load rate changed if materials have shown different mechanical behavior characteristics: i.e., weak or ductile. Group C specimens showed weaker behavior than those of group B, as indicated by the sharp increase in hits and amplitude. In terms of duration of the tests, this is explained by the fewer number of seconds at which delamination occurred relative to the group B specimens.

AE allows the detection of a critical stage by the large increment in hit numbers and amplitude many seconds before the dashed line. This predictive capability is more evident if the extrusion temperature is higher; samples in group C display the greatest increase in slope, providing confirmation of higher acoustic activity compared to the other groups. This advantage has also been noted for several other materials [27,28,29,30,31,32,33], and is very important for FDM parts, given that, in most cases, delamination occurs internally and is not visible.

## 4. Conclusions

This paper presents the results of Mode I delamination tests, monitored by acoustic emissions, performed on fused deposition modelling parts extruded at three different temperatures (220 °C, 230 °C, and 240 °C). It was observed that if extrusion temperature is increased, delamination is reduced and *G_I_* toughness is increased. Acoustic emission analysis provided relevant predictive information about the material under testing. In particular, higher acoustic activity combined with a higher extrusion temperature provides a more predictable signal of delamination than any visual indication.

## Figures and Tables

**Table 1:** Specimen dimensions according to ASTM D5528.

Specimen Dimension	Value in Standard (mm)	Chosen Value (mm)
L_min_ (length)	125	125
b (width)	20 … 25	25
h (height)	3 … 5 (variation ≤ 0.1)	3
a (crack)	63	63
a_0_ (initial crack)	50	50

**Table 2:** Main printing settings.

Parameter	Value
Air gap (mm)	0
Layer thickness (mm)	0.35
Bead width (mm)	0.70
Number of contour lines	3
